# Nomogram for predicting inadequate treatment response in vertebral osteomyelitis

**DOI:** 10.1080/07853890.2026.2648906

**Published:** 2026-03-31

**Authors:** Jianlong Li, Yingxin Zhao, Liang Xu, Yongrui Yang, Wenkai Ruan, Rongpan Dang, Wentao Zhao, Huigang An, Hongdong Tan

**Affiliations:** Shandong Public Health Clinical Center, Shandong University, Shandong, China

**Keywords:** Vertebral osteomyelitis, nomogram, inadequate treatment response, prediction model

## Abstract

**Background:**

Vertebral osteomyelitis (VO) is frequently complicated by inadequate treatment response (ITR), resulting in prolonged hospitalisation and recurrence. No validated tool currently exists for early ITR risk prediction.

**Purpose:**

To develop and internally validate a nomogram for predicting ITR probability in patients with VO.

**Materials and Methods:**

This retrospective study consecutively enrolled 219 patients with vertebral osteomyelitis who were admitted to the Department of Orthopaedics at Shandong Public Health Clinical Centre between January 1, 2021, and December 31, 2024; all data were extracted and analysed in October 2025.ITR was defined as clinical, microbiological, or radiological progression despite ≥4 weeks of appropriate antimicrobial therapy. Predictors were selected using LASSO regression and multivariate logistic regression. Nomogram performance was assessed by AUC, calibration curve, Hosmer-Lemeshow test, decision curve analysis (DCA), and 1000 bootstrap resamples for internal validation.

**Results:**

Fifty-one patients (23.3%) developed ITR within 6 months. Six independent predictors were identified: intraspinal abscess, paraspinal abscess, spinal implant, concomitant pneumonia, low prealbumin, and multiple-site infections. The nomogram yielded excellent discrimination (AUC 0.909, 95% CI 0.862–0.956), confirmed by bootstrap validation (AUC 0.91, 95% CI 0.85–0.95). At the optimal cutoff of 0.308, sensitivity was 76.5%, specificity 91%, Positive Predictive Value (PPV) 70%, and Negative Predictive Value (NPV) 93.2%. Calibration was satisfactory (Hosmer-Lemeshow *p* = 0.750), and DCA demonstrated clear clinical net benefit.

**Conclusion:**

We developed the first validated nomogram for individualised ITR risk prediction in VO, enabling early therapeutic intensification and potentially improving clinical outcomes.

## Introduction

Spinal infections, including vertebral osteomyelitis, discitis, and epidural abscesses, pose a formidable clinical challenge due to the high prevalence of inadequate treatment response (ITR), ITR, characterized by clinical, microbiological, or radiological progression despite treatment, is influenced by delayed diagnosis, antimicrobial resistance, and patient comorbidities like diabetes or immunosuppression [1,2] which occurs in 20-40% of cases and can lead to devastating consequences if not addressed promptly [3,4]. These infections, commonly caused by bacterial pathogens such as Staphylococcus aureus or Escherichia coli, often result in persistent symptoms, sepsis, recurrence, neurological deficits, and even mortality when initial antimicrobial therapy fails, exacerbating prolonged hospitalization and healthcare burdens [5–7]. Accurate prediction of treatment response is essential for optimizing clinical decision-making, enabling timely therapeutic adjustments (surgical debridement or regimen changes), and mitigating complications. By identifying high-risk patients, clinicians can escalate interventions, allocate resources efficiently, and enhance long-term outcomes, ultimately reducing the substantial economic and quality-of-life impacts of chronic spinal morbidity [8,9].

Despite the clinical importance of treatment response prediction, current research on prognostic models for vertebral osteomyelitis remains sparse and methodologically inconsistent. Traditional monitoring approaches, such as serial inflammatory markers (CRP/ESR) or imaging follow-up, lack specificity and often fail to integrate multifaceted risk factors, such as microbiological profiles or comorbidity burden [10]. Recent studies have explored risk factors for poor treatment response in vertebral osteomyelitis, identifying variables such as previous surgery, severe sepsis, and elevated inflammatory markers as significant predictors [1,6,11]. However, these studies often lack external validation and rarely integrate multiple risk factors into a cohesive predictive framework. Furthermore, machine learning-based models, while promising, often suffer from poor interpretability and generalizability, limiting their clinical utility [12]. These shortcomings highlight the urgent need for a reliable, user-friendly tool that can integrate diverse clinical variables to predict treatment response accurately in patients with vertebral osteomyelitis.

Nomograms offer a promising solution, providing visual, intuitive risk quantification from multiple predictors, combining statistical rigour with bedside applicability, unlike traditional monitoring or machine learning models [13]. Successfully used in oncology and infectious diseases to predict treatment failure [14], they could integrate demographics, biomarkers, imaging, and treatment data for personalised inadequate response predictions in vertebral osteomyelitis, enhancing risk stratification and guiding interventions like extended antibiotics or surgery. Recent studies show nomograms’ efficacy in sepsis and postoperative infections, suggesting applicability to vertebral osteomyelitis [15,16]. However, no validated nomogram exists for predicting inadequate treatment response in vertebral osteomyelitis, a critical literature gap.

This study aims to develop and validate a nomogram for predicting inadequate treatment response (clinical, microbiological, or radiological progression within six months) in patients with vertebral osteomyelitis, leveraging a comprehensive set of clinical, laboratory, and imaging variables.

## Materials and methods

### Study design

**Figure 1.**
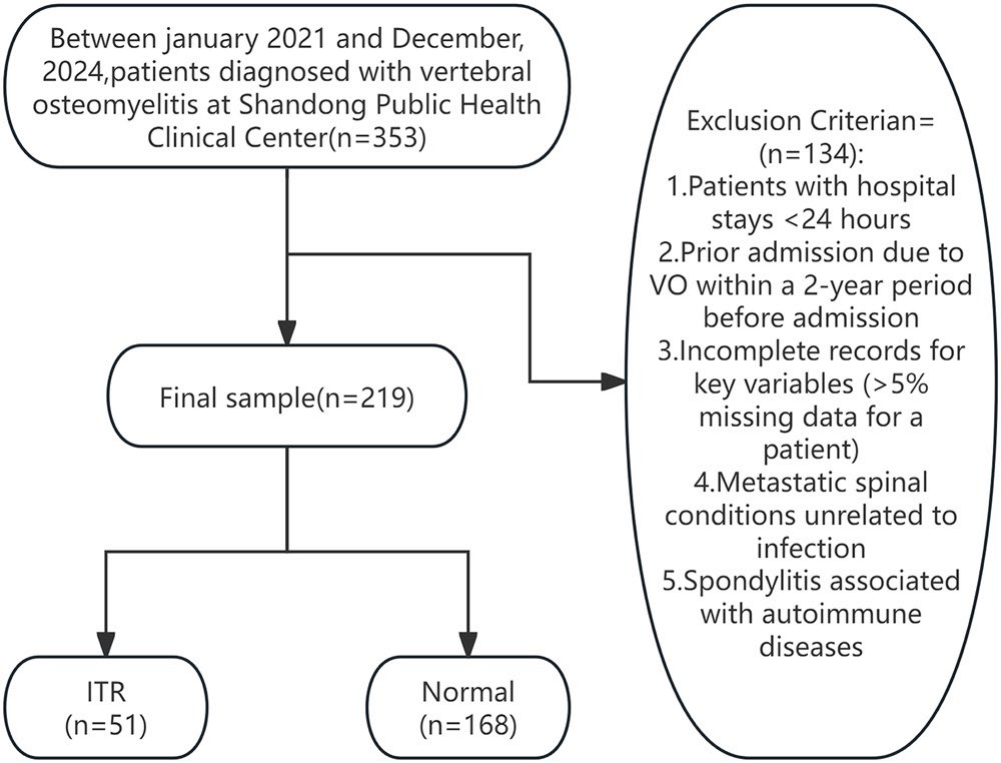
An illustration of the study flow chart.

This was a single-centre retrospective cohort study conducted to develop and internally validate a predictive model for inadequate treatment response (ITR) in patients with vertebral osteomyelitis (including both hematogenous and postoperative infections). Consecutive patients admitted to the Department of Orthopaedics at Shandong Public Health Clinical Centre between January 1, 2021, and December 31, 2024, were included. All clinical, laboratory, and imaging data were retrieved from the electronic medical record system, and the database was finalised and statistically analysed in October 2025. (Figure 1)

The primary outcome was inadequate treatment response (ITR) within 6 months after diagnosis, defined as clinical, microbiological, or radiological progression despite ≥4 weeks of appropriate antimicrobial therapy. This operational definition aligns with previous studies on vertebral osteomyelitis that use similar criteria to identify treatment failure or poor response, including persistent/worsening symptoms, failure of inflammatory markers, radiological progression (enlarging abscess or bone destruction), or microbiological evidence of ongoing infection after adequate antibiotic exposure [1,2,5,6,17]

The study was performed in accordance with the Declaration of Helsinki and adhered to the Transparent Reporting of a Multivariable Prediction Model for Individual Prognosis or Diagnosis (TRIPOD) statement (type 2b) [18]. The study protocol was approved by the Institutional Review Board of Shandong Public Health Clinical Centre (Approval No. [GWLCZXEC-SOP-K-2025-137]; date of approval: [August 28, 2025]). Owing to the retrospective design and use of anonymised data, the requirement for written informed consent was waived by the ethics committee.

### Patient population and selection criteria

The inclusion criteria for this study were as follows: Adult patients (≥18 years) diagnosed with primary or secondary including pyogenic spondylodiscitis, tuberculous spondylitis, brucellar spondylitis, fungal spondylitis, infectious arthritis of the spinal facet joints, multifocal spinal infections, spinal epidural abscess, and spinal implant-related infection (corresponding ICD-10 codes: M46.1, M46.4, M46.5, M48.0, A23.3, M49.1, G06.1, T84.63);hospitalised in the orthopedics department; complete follow-up data available for at least 6 months after diagnosis. Exclusion Criteria: Patients with hospital stays <24 h; prior admission due to VO within a 2-year period before admission, incomplete records for key variables (>5% missing data for a patient); metastatic spinal conditions unrelated to infection; spondylitis associated with autoimmune diseases.

The primary outcome was inadequate treatment response (ITR) within 6 months after diagnosis, defined as clinical, microbiological, or radiological progression despite ≥4 weeks of antimicrobial therapy, determined through retrospective review of hospital records and follow-up data, supplemented by confirmation from imaging reports or repeat cultures.

### Data collection

Data were collected from electronic medical records, including admission notes, laboratory results, imaging reports, surgical records, and follow-up documentation. Variables were selected based on clinical relevance and established risk factors for inadequate treatment response (ITR) from prior literature on vertebral osteomyelitis. The variables included in the analysis were: Etiology, Intraspinal abscess, Paraspinal abscess, Multi-segment involvement, Skip lesion, Multi-drug resistant organisms, Implant presence, Pneumonia, Age, Sex, Disease duration (time from symptom onset to diagnosis), Nerve injury (spinal cord involvement), Hypertension, Diabetes, Immunosuppressive disease, Chronic liver disease, Kidney disease, Septicopyemia, Affected vertebral segment, White blood cell count (WBC), Red blood cell count (RBC), Hemoglobin (Hb), Platelet count (Plt), C-reactive protein (CRP), Erythrocyte sedimentation rate (ESR), Total protein, Albumin, Prealbumin (PLAB), and Interleukin-6 (IL-6).

### Statistical methods

This study followed the TRIPOD reporting guidelines [18]. Statistical analyses were performed using R software (version 4.2.2; R Foundation for Statistical Computing, Vienna, Austria) and the Free Statistics Analysis Platform (version 2.2, Beijing, China). Data from Shandong Provincial Public Health Clinical Centre were used for model development, and the performance of the model was internally validated using bootstrap resampling. Continuous variables were expressed as mean (SD) or median (IQR) and compared using t-tests or Wilcoxon rank-sum tests, as appropriate. Categorical variables were summarised as frequencies and percentages and compared using the Chi-square test or Fisher’s exact test.

### Variable selection and prediction model establishment

A logistic univariate analysis was conducted to screen out the potential risk factors for ITR in the training cohort. Variables with a *p* value less than 0.05 in the logistic univariate analysis and those with significant clinical importance were considered as potential candidate factors for constructing the nomogram. Then, we employed the Least Absolute Shrinkage and Selection Operator (LASSO) regression, a robust technique for high-dimensional prediction, to pinpoint potential predictive variables for patients. The selected variables were subjected to binary logistic regression analysis to construct a nomogram.

### Model validation and performance evaluation

The predictive performance of the model was comprehensively evaluated in terms of discrimination, calibration, and clinical utility. Discrimination was assessed using the area under the receiver operating characteristic curve (AUC). Calibration was evaluated by calibration plots and the Hosmer–Lemeshow goodness-of-fit test, with a non-significant result suggesting satisfactory agreement between predicted and observed outcomes [19]. The optimal cutoff value of the nomogram was determined by maximising the Youden index (sensitivity + specificity − 1), and corresponding sensitivity, specificity, and accuracy were calculated. Internal validation was performed using bootstrap resampling with 1000 iterations, without splitting the dataset into separate training and testing sets. This approach follows TRIPOD recommendations [18] and is preferred over data splitting in moderate-sized cohorts, as it uses the full sample (*n* = 219) for model development while providing stable, optimism-corrected estimates of performance and accounting for variability in predictor selection (*via* LASSO) and logistic regression fitting. In each bootstrap iteration, a sample of size *n* = 219 was drawn with replacement from the original dataset; the entire modelling process (LASSO selection + multivariable logistic regression) was repeated on the bootstrap sample; and the resulting model was applied to the original dataset to compute performance metrics. The final optimism-corrected AUC (and other metrics) was obtained by averaging across the 1000 bootstraps, thereby mitigating overfitting.

Clinical utility was further examined using decision curve analysis (DCA), which quantifies the net benefit across a range of threshold probabilities [20]. A two-sided *p*-value <0.05 was considered statistically significant.

## Results

### General characteristics

Between January 2021 and December 2024, a total of 353 patients with vertebral osteomyelitis were retrospectively screened, of whom 219 (62%) met the eligibility criteria at Shandong Provincial Public Health Clinical Centre, Shandong University. The demographic and clinical characteristics of these patients are summarised in Table 1. The mean age was 59.8 years, with 61.6% male patients. The causative pathogen could not be identified in 42 cases. Among the remaining patients, 34 were infected with Mycobacterium tuberculosis, 27 with Brucella species, 111 with Pyogenic bacteria, and 5 with fungi. Overall, 29.7% of patients were infected with multidrug-resistant (MDR) organisms, and 13.7% of infections were associated with indwelling implants. (Table 1)

**Table 1.** The baseline characteristics group of ITR and normal conditions.

Variables	Total (*n* = 219)	No (*n* = 168)	Yes (*n* = 51)	*p*
Pathogenesis *n* (%)				0.036
Not identified	42 (19.2)	36 (21.4)	6 (11.8)	
Tubercle	34 (15.5)	24 (14.3)	10 (19.6)	
Brucella	27 (12.3)	22 (13.1)	5 (9.8)	
Pyogenic	111 (50.7)	85 (50.6)	26 (51)	
Fungus	5 (2.3)	1 (0.6)	4 (7.8)	
Intraspinal abscess *n* (%)				<0.001
No	156 (71.2)	137 (81.5)	19 (37.3)	
Yes	63 (28.8)	31 (18.5)	32 (62.7)	
Paraspinal abscess *n* (%)				<0.001
No	160 (73.1)	140 (83.3)	20 (39.2)	
Yes	59 (26.9)	28 (16.7)	31 (60.8)	
Multiple segment *n* (%)				<0.001
No	165 (75.3)	143 (85.1)	22 (43.1)	
Yes	54 (24.7)	25 (14.9)	29 (56.9)	
Skip lesion *n* (%)				0.092
No	199 (90.9)	156 (92.9)	43 (84.3)	
Yes	20 (9.1)	12 (7.1)	8 (15.7)	
Multi-drugresistant *n* (%)				0.04
No	154 (70.3)	124 (73.8)	30 (58.8)	
Yes	65 (29.7)	44 (26.2)	21 (41.2)	
Spinal implant *n* (%)				<0.001
No	189 (86.3)	156 (92.9)	33 (64.7)	
Yes	30 (13.7)	12 (7.1)	18 (35.3)	
Pneumonia *n* (%)				<0.001
No	186 (84.9)	157 (93.5)	29 (56.9)	
Yes	33 (15.1)	11 (6.5)	22 (43.1)	
Age Mean ± SD	59.8 ± 15.3	58.8 ± 15.5	63.2 ± 14.1	0.072
Sex *n* (%)				0.258
Female	84 (38.4)	61 (36.3)	23 (45.1)	
Male	135 (61.6)	107 (63.7)	28 (54.9)	
Disease time Median (IQR)	40.0 (22.5, 90.0)	60.0 (20.8, 90.0)	30.0 (30.0, 67.5)	0.227
Neurotrauma *n* (%)				0.132
No	170 (77.6)	135 (80.4)	35 (68.6)	
Yes	49 (22.4)	33 (19.6)	16 (31.4)	
Hypertension *n* (%)				0.547
No	174 (79.5)	135 (80.4)	39 (76.5)	
Yes	45 (20.5)	33 (19.6)	12 (23.5)	
Diabetes *n* (%)				0.013
No	180 (82.2)	144 (85.7)	36 (70.6)	
Yes	39 (17.8)	24 (14.3)	15 (29.4)	
Immunosuppressive diseases *n* (%)				0.243
No	208 (95.4)	162 (96.4)	46 (92)	
Yes	10 (4.6)	6 (3.6)	4 (8)	
Hepatopathy *n* (%)				1
No	213 (97.3)	163 (97)	50 (98)	
Yes	6 (2.7)	5 (3)	1 (2)	
Nephropathy *n* (%)				0.392
No	211 (96.3)	163 (97)	48 (94.1)	
Yes	8 (3.7)	5 (3)	3 (5.9)	
Septicopyemia *n* (%)				0.002
No	175 (79.9)	142 (84.5)	33 (64.7)	
Yes	44 (20.1)	26 (15.5)	18 (35.3)	
Lesioned.segment *n* (%)				0.67
Cervical	9 (4.1)	6 (3.6)	3 (6.1)	
Thoracic	35 (16.1)	26 (15.5)	9 (18.4)	
Lumbar	170 (78.3)	133 (79.2)	37 (75.5)	
Sacral	3 (1.4)	3 (1.8)	0 (0)	
WBC Mean ± SD	7.0 ± 3.0	6.8 ± 2.8	7.6 ± 3.8	0.095
RBC Mean ± SD	4.1 ± 2.2	4.2 ± 2.4	3.9 ± 1.1	0.393
Hb Mean ± SD	112.8 ± 19.1	115.9 ± 18.3	102.4 ± 18.1	<0.001
Plt Mean ± SD	280.8 ± 115.1	281.1 ± 118.2	280.0 ± 105.8	0.952
CRP Median (IQR)	38.0 (13.4, 68.2)	29.1 (8.8, 57.7)	58.5 (34.7, 98.1)	<0.001
ESR Mean ± SD	52.5 ± 32.0	50.3 ± 31.2	59.6 ± 34.2	0.069
Total protein Mean ± SD	65.7 ± 7.3	66.2 ± 6.8	64.0 ± 8.4	0.058
Albumin Mean ± SD	36.0 ± 4.6	36.4 ± 4.7	34.8 ± 4.2	0.032
Prealbumin Mean ± SD	152.1 ± 66.5	167.3 ± 62.0	102.3 ± 55.7	<0.001

### Identification of predictive factors

**Figure 2.**
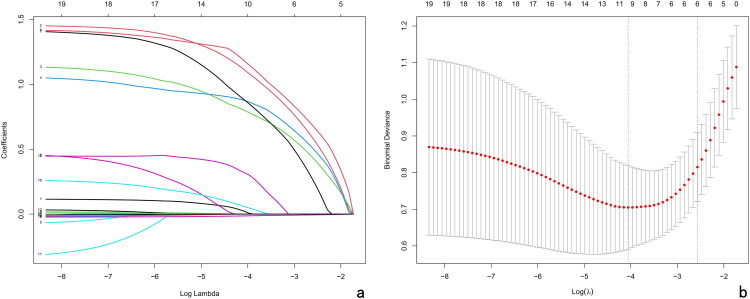
Feature selection using LASSO binary logistic regression model. (a) LASSO coefficient profiles. The plot displays the coefficient paths of 19 candidate features as a function of the log(λ) penalty parameter. A coefficient profile is generated against the log(λ) sequence, with non-zero coefficients retained as λ decreases. (b) Optimal λ selection *via* five-fold cross-validation. The partial likelihood deviance (binomial deviance) is plotted against log(λ), together with its standard error. Two vertical dashed lines indicate the λ value that minimises the deviance (λmin) and the largest λ within one standard error of the minimum (λ1SE). The λ1SE criterion was applied, yielding 6 non-zero coefficients. LASSO, least absolute shrinkage and selection operator.

In the training cohort, 27 clinical and imaging indicators were initially collected for each patient. Univariate logistic regression identified 19 variables (Etiology, Intraspinal abscess, Paraspinal abscess, Multi segment, Skip lesion, Multi drug resistant, Implant, Pneumonia, Age, Diabetes, Septicopyemia, WBC, Hb, CRP, ESR, Total Protein, Albumin, Prealbumin)significantly associated with inadequate treatment response (ITR). To minimize noise, only these significant variables were carried forward into the LASSO regression analysis. LASSO regression was then applied to refine the predictors, reducing the risk of overfitting and enhancing model robustness. Using the 1-SE criterion, six key variables were ultimately retained (Figure 2), including paraspinal abscess, intraspinal abscess, indwelling implants, pneumonia, multifocal infection, and prealbumin levels.

### Development and assessment of the predictive nomogram

**Figure 3.**
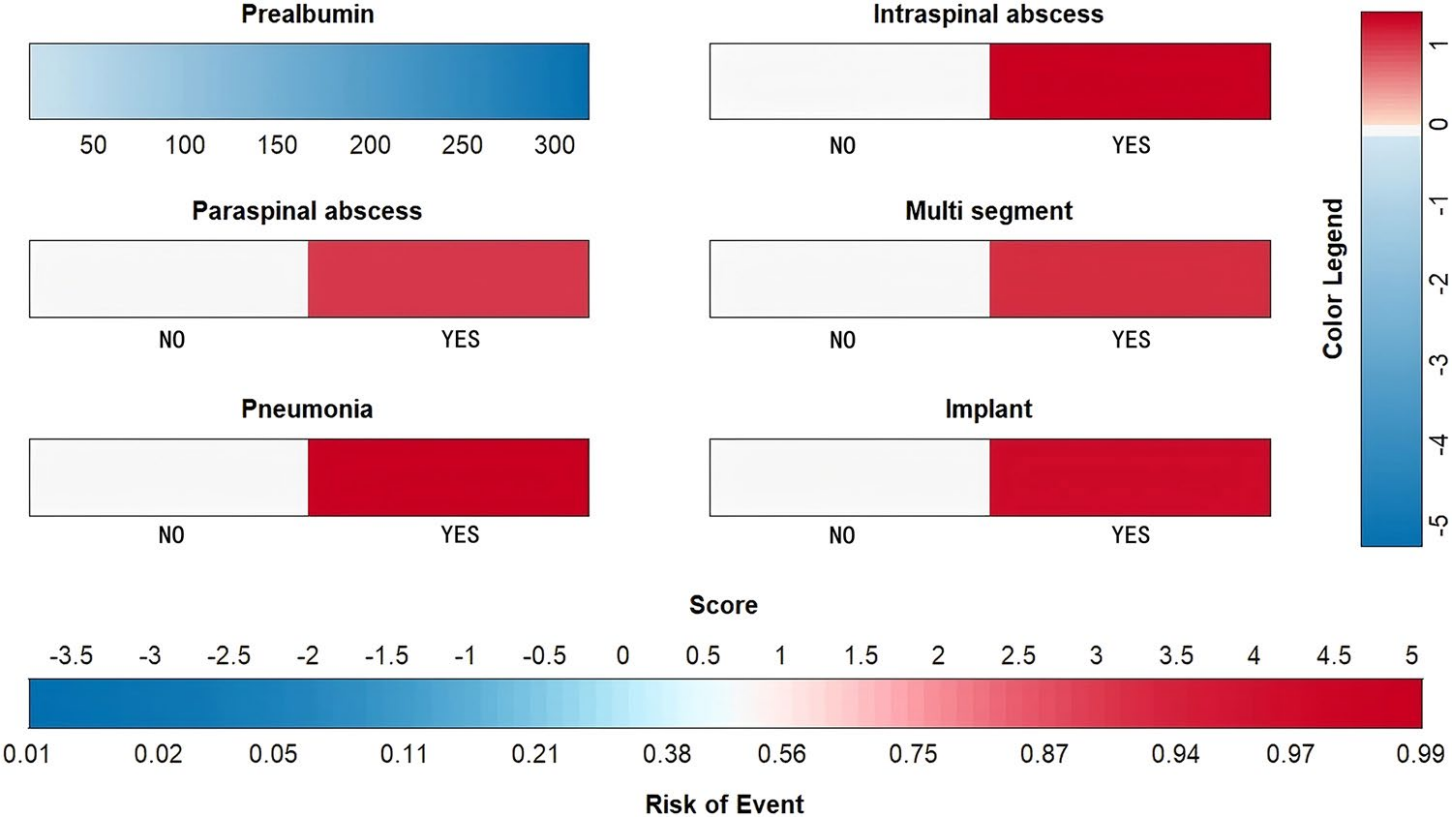
Nomogram for ITR projection in vertebral osteomyelitis.

Based on significant variables identified by logistic regression and nonzero coefficients selected through LASSO regression, a prognostic nomogram was developed incorporating the following predictors: paraspinal abscess, intraspinal abscess, implants, pneumonia, multifocal infection, and prealbumin levels. (Table 2) In the nomogram, each predictor was assigned a weighted score, and the total score for an individual patient corresponded to the estimated probability of ITR within 6 months. (Figure 3)

**Table 2.** Multivariate regression model based on LASSO regression results.

Predictor Variable	β	SE	OR	CI	*p*
(Intercept)	−0.757	0.58015	0.469	0.469 (0.150–1.462)	0.192
Intraspinal abscess	1.371	0.48474	3.94	3.940 (1.523–10.187)	<0.01
Paraspinal abscess	1.007	0.50101	2.738	2.738 (1.026–7.311)	<0.05
Implant	1.281	0.6183	3.6	3.600 (1.072–12.095)	<0.05
Pneumonia	1.373	0.55109	3.948	3.948 (1.341–11.628)	<0.05
Prealbumin	−0.016	0.00403	0.984	0.984 (0.976–0.991)	<0.001
Multi segment	1.096	0.4925	2.991	2.991 (1.139–7.853)	<0.05

**Figure 4.**
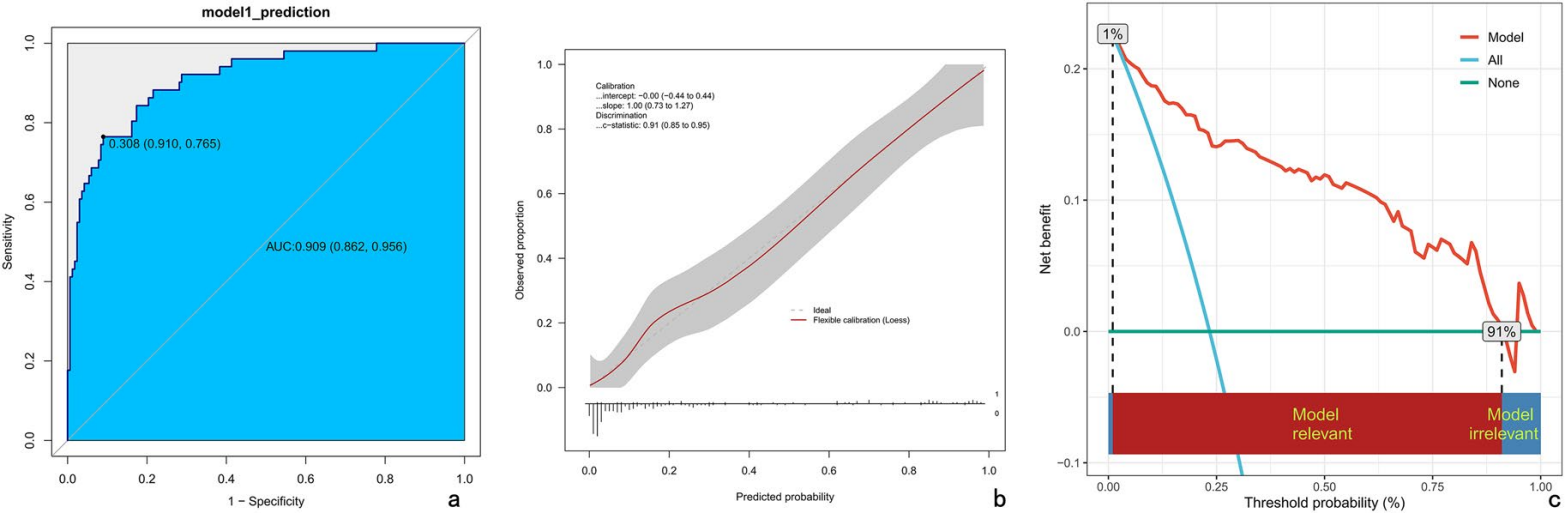
(a) Receiver Operating Characteristic (ROC) Curve of the Prognostic Nomogram. The marked point on the curve indicates the optimal cutoff value, corresponding to its specificity and sensitivity. The 95% confidence interval for the area under the curve (AUC) is provided in parentheses. (b) Calibration Curve of the Nomogram. The apparent curve illustrates the association between predicted and observed probabilities of Inadequate Treatment Response (ITR) within 6 months, with the bias-corrected curve derived from 1000 bootstrap resamples. The ideal curve, a 45° line, represents perfect predictive accuracy. (c) Decision Curve Analysis (DCA) of the Nomogram. The red solid line depicts the nomogram’s net benefit, with the x-axis showing threshold probabilities and the y-axis indicating net benefit. AUC, area under the curve; ROC, receiver operating characteristic.

The area under the receiver operating characteristic curve (AUC) was 0.909 (95% CI: 0.862–0.956), and the internally validated AUC was (AUC:0.91, 95% CI 0.85–0.95). The optimal cutoff value selected as a predictive threshold was 0.308, with a sensitivity of 76.5%, specificity of 91%, positive predictive value of 70%, negative predictive value of 93.2%, and accuracy of 91.3%. The Hosmer-Lemeshow test yielded a P value of 0.75. The calibration plot exhibited high consistency in prediction, and decision curve analysis showed excellent net benefits. (Figure 4)

## Discussion

In our multivariable framework, six variables—paraspinal abscess, intraspinal abscess, indwelling implants, pneumonia, multiple segments, and lower prealbumin level—emerged as independent high-risk factors for inadequate treatment response (ITR) in patients with vertebral osteomyelitis. These predictors were incorporated into our prognostic nomogram, which demonstrated excellent discriminative ability with an AUC of 0.909 and robust calibration, providing a reliable tool for early risk stratification. The identification of these variables aligns with established risk factors for ITR in vertebral osteomyelitis, reflecting the complex interplay of infection extension, systemic complications, and nutritional status.

Paraspinal abscess was a significant predictor of ITR in our model, consistent with prior studies [5,21,22] linking undrained abscesses to persistent infection and treatment failure in vertebral osteomyelitis due to incomplete pathogen clearance and heightened sepsis risk. For instance, studies have shown that paraspinal abscesses are associated with higher relapse rates and prolonged therapy needs, as evidenced in cohorts where surgical drainage significantly improves response rates compared to paraspinal abscesses treated with antibiotics alone [22].

Intraspinal abscess, another key factor in our nomogram, likely reflects advanced infection spread, amplifying ITR risk through neurological compromise and persistent inflammation, particularly in cases with epidural involvement where abscesses lead to treatment failure in up to 30% of patients [5,11,23,24]. Research indicates that intraspinal abscesses complicate the course of vertebral osteomyelitis, with early predictors like immunosuppression elevating its risk and contributing to higher overall ITR [23,24].

Indwelling implants, including spinal hardware or prostheses, underscore the role of biofilm formation in exacerbating poor outcomes, as these patients are less responsive to antibiotics and face increased risks of chronic infection [6,25,26]. Studies suggest that implant-related infections are a leading cause of ITR in postoperative spinal cases, occurring with higher failure rates in patients with pre-existing hardware undergoing treatment [27–28].

Pneumonia, a concurrent complication in our model, is associated with systemic inflammation and ITR, with prior literature confirming its link to treatment failure in vertebral osteomyelitis, where respiratory involvement often exceeds 20% of cases [29,30]. Multivariate analyses have revealed that pneumonia at admission correlates with poorer response, while it is linked to higher comorbidity burdens and 6-month failure rates in affected patients [4,31].

Multifocal vertebral osteomyelitis indicates a greater disease burden, complicating treatment and correlating with ITR, as seen in studies where multifocal involvement increases the likelihood of persistent infection, leading to higher failure rates [32–34]. Factors like delayed debridement and immunocompromise have been identified as independent risks for multifocal progression in vertebral osteomyelitis, with overall rates contributing to extended therapy needs [21,35].

Lower prealbumin levels capture nutritional deficiency, reinforcing the role of malnutrition in ITR risk, with studies showing that low prealbumin (<20 mg/dL) predicts prolonged infection and poorer response in vertebral osteomyelitis cases [36,37]. Research emphasises that nutritional support is critical in undernourished patients, where low prealbumin levels are associated with elevated ITR and slower recovery [38,39].

Although previous studies have indicated that age and liver dysfunction, and renal dysfunction are risk factors for adverse outcomes—including inadequate treatment response (ITR), recurrence, and mortality in vertebral osteomyelitis, these variables were not independently associated in our model following multivariable adjustment [31,40,41].

First,cohort-specific differences likely explain this: our single-centre orthopaedic population at Shandong Public Health Clinical Centre had a low prevalence of nephropathy (3.7%, Table 1; univariate *p* = 0.392) and presumably milder or lower rates of severe liver disease and advanced age-related comorbidity compared to cohorts with more critically ill or dialysis-dependent patients [40,41]. Second, our endpoint was specifically inadequate treatment response (ITR) during antimicrobial therapy (progression despite ≥4 weeks of appropriate antibiotics), whereas renal dysfunction more strongly influences broader outcomes like mortality, sepsis, or recurrence *via* impaired drug clearance and systemic effects [17,31,42,43]. Third, these factors may have been subsumed by dominant predictors in the model (low prealbumin, abscesses, implants, pneumonia), resulting in multicollinearity and loss of independent significance [44]. Their exclusion does not negate their clinical importance; renal function and comorbidities should still be monitored, as they may indirectly affect ITR through nutritional status or treatment tolerance. Multicenter external validation is needed to confirm these findings across diverse populations.

Several studies have explored risk factors and prediction models for adverse outcomes in vertebral osteomyelitis, but none have developed a validated nomogram specifically for inadequate treatment response (ITR) as defined in our study. For example, a nationwide cohort of 2148 patients identified factors associated with recurrence after early spinal instrumentation for pyogenic spondylodiscitis, including older age, posterior thoracic approach, multiple approaches, cage use, transfusion, resistant organisms, and prolonged steroids, with multivariable models showing AUC =0.813 [45]. Other models predicted recurrence using time-series CRP data *via* artificial neural networks (sensitivity improved to 55.8%–60.5% when incorporating treatment response) [46]. Prognostic nomograms have been developed for 1-year mortality in severe osteomyelitis (including vertebral cases), incorporating comorbidities, sepsis, and CRP trends (AUC 0.74–0.876) [2,47]. Multifactorial analyses identified predictors of treatment failure or poor outcome, such as diabetes, neurological impairment, epidural abscess, longer symptom duration, high CRP, S. aureus infection, and motor weakness [6,46,48]. These prior efforts often focus on different endpoints (recurrence or mortality rather than early ITR during antimicrobial therapy), lack visual nomograms for clinical use, or do not combine LASSO variable selection with bootstrap validation.

Compared to prior studies, our research demonstrates several notable strengths. First, the high authenticity and consistency of our data derive from real-world clinical records at a single institution, ensuring uniformity in diagnostic processes and enhancing data integrity and accuracy. Second, our dataset encompasses comprehensive clinical information, extending beyond basic demographic characteristics to include detailed comorbidities, immediate clinical presentations upon admission, extensive laboratory results, and in-depth imaging features, providing a robust foundation for prognostic modeling. Third, we employed bootstrap internal validation to rigorously assess the model’s discrimination and calibration, confirming its robustness and reliability with an AUC of 0.909.

Despite these strengths, several limitations must be acknowledged. First, the retrospective design introduces the potential for selection bias, which cannot be fully mitigated. Second, as a single-centre study conducted at Shandong Public Health Clinical Centre, and a lack of external validation, the generalizability of our findings may be limited, particularly in populations with differing epidemiological profiles. Conducting multi-centre external validation in future studies would be essential to further confirm the robustness and broader clinical applicability of our nomogram. Finally, although our sample size was sufficient for model development, the number of variables evaluated relative to the number of primary outcome events may increase the risk of overfitting, potentially impacting the model’s predictive accuracy.

## Conclusion

This study represents the first attempt to develop and validate a predictive model for inadequate treatment response risk in patients with vertebral osteomyelitis. This model enables clinicians to determine individualised response probabilities, thereby facilitating timely therapeutic modifications and improved clinical management.

## Data Availability

The datasets used and/or analysed during the current study are available from the corresponding author on reasonable request.

## References

[1] Geisler Crone C, Mose Tetens M, Bengaard Andersen A, et al. Clinical characteristics of pyogenic vertebral osteomyelitis, and factors associated with inadequate treatment response. Int J Infect Dis. 2021;108:487–493. doi: 10.1016/j.ijid.2021.05.078. 34091001

[2] Widdrington JD, Emmerson I, Cullinan M, et al. Pyogenic spondylodiscitis: risk factors for adverse clinical outcome in routine clinical practice. Med Sci (Basel). 2018;6(4):96. doi: 10.3390/medsci6040096. 30380776 PMC6313505

[3] Herren C, Jung N, Pishnamaz M, et al. Spondylodiscitis: diagnosis and treatment options. Dtsch Arztebl Int. 2017;114(51-52):875–882. doi: 10.3238/arztebl.2017.0875. 29321098 PMC5769318

[4] Gupta A, Kowalski TJ, Osmon DR, et al. Long-term outcome of pyogenic vertebral osteomyelitis: a cohort study of 260 patients. Open Forum Infect Dis. 2014;1(3):ofu107. doi: 10.1093/ofid/ofu107. 25734175 PMC4324221

[5] Berbari, E. F., Kanj, S. S., Kowalski, T. J., Darouiche, R. O., Widmer, A. F., Schmitt, S. K., Hendershot, E. F., Holtom, P. D., Huddleston, P. M., 3rd, Petermann, G. W., Osmon, D. R., & Infectious Diseases Society of America (2015). 2015 infectious diseases society of America (IDSA) clinical practice guidelines for the diagnosis and treatment of native vertebral osteomyelitis in adults. Clin Infect Dis, 61(6), e26–e46. doi: 10.1093/cid/civ482. 26229122

[6] Arnold R, Rock C, Croft L, et al. Factors associated with treatment failure in vertebral osteomyelitis requiring spinal instrumentation. Antimicrob Agents Chemother. 2014;58(2):880–884. doi: 10.1128/AAC.01452-13. 24277039 PMC3910840

[7] Yagdiran A, Otto-Lambertz C, Lingscheid KM, et al. Quality of life and mortality after surgical treatment for vertebral osteomyelitis (VO): a prospective study. Eur Spine J. 2021;30(6):1721–1731. doi: 10.1007/s00586-020-06519-z. 32613398

[8] Yagdiran A, Bredow J, Weber C, et al. the burden of vertebral osteomyelitis-an analysis of the workforce before and after treatment. J Clin Med. 2022;11(4):1095. doi: 10.3390/jcm11041095. 35207367 PMC8875884

[9] Dragsted C, Aagaard T, Ohrt-Nissen S, et al. Mortality and health-related quality of life in patients surgically treated for spondylodiscitis. J Orthop Surg (Hong Kong). 2017;25(2):2309499017716068. doi: 10.1177/2309499017716068. 28639530

[10] Yoon SH, Chung SK, Kim KJ, et al. Pyogenic vertebral osteomyelitis: identification of microorganism and laboratory markers used to predict clinical outcome. Eur Spine J. 2010;19(4):575–582. doi: 10.1007/s00586-009-1216-1. 19937064 PMC2899831

[11] Jung N, Ernst A, Joost I, et al. Vertebral osteomyelitis in patients with Staphylococcus aureus bloodstream infection: evaluation of risk factors for treatment failure. J Infect. 2021;83(3):314–320. doi: 10.1016/j.jinf.2021.06.010. 34146597

[12] Lee J, Ruiz-Cardozo MA, Patel RP, et al. Clinical prediction for surgical versus nonsurgical interventions in patients with vertebral osteomyelitis and discitis. J Spine Surg. 2024;10(2):204–213. doi: 10.21037/jss-23-111. 38974494 PMC11224782

[13] Balachandran VP, Gonen M, Smith JJ, et al. Nomograms in oncology: more than meets the eye. Lancet Oncol. 2015;16(4):e173–e180. doi: 10.1016/S1470-2045(14)71116-7. 25846097 PMC4465353

[14] Yu ZJ, Dou Z, Li J, et al. Nomogram for predicting in-hospital mortality in infective endocarditis based on early clinical features and treatment options. Front Cardiovasc Med. 2022;9:882869. doi: 10.3389/fcvm.2022.882869. 35571168 PMC9091715

[15] Ren Y, Zhang L, Xu F, et al. Risk factor analysis and nomogram for predicting in-hospital mortality in ICU patients with sepsis and lung infection. BMC Pulm Med. 2022;22(1):17. doi: 10.1186/s12890-021-01809-8. 34991569 PMC8739695

[16] Huang X, Guo Y, Fu R, et al. A nomogram to predict postoperative surgical site infection of adult patients who received orthopaedic surgery: a retrospective study. Sci Rep. 2023;13(1):8129. doi: 10.1038/s41598-023-34926-x. 37208366 PMC10199048

[17] Park KH, Cho OH, Lee JH, et al. Optimal duration of antibiotic therapy in patients with hematogenous vertebral osteomyelitis at low risk and high risk of recurrence. Clin Infect Dis. 2016;62(10):1262–1269. doi: 10.1093/cid/ciw098. 26917813

[18] Collins GS, Reitsma JB, Altman DG, et al. Transparent reporting of a multivariable prediction model for individual prognosis or diagnosis (TRIPOD): the TRIPOD statement. BMJ. 2015;350:g7594. doi: 10.1136/bmj.g7594. 25569120

[19] Lemeshow S, Hosmer DW. Jr A review of goodness of fit statistics for use in the development of logistic regression models. Am J Epidemiol. 1982;115(1):92–106. doi: 10.1093/oxfordjournals.aje.a113284. 7055134

[20] Vickers AJ, Elkin EB. Decision curve analysis: a novel method for evaluating prediction models. Med Decis Making. 2006;26(6):565–574. doi: 10.1177/0272989X06295361. 17099194 PMC2577036

[21] Matsuo T, Borgonovo F, Lahr BD, et al. Clinical manifestations, long-term trends, and risk factors for treatment failure in native vertebral osteomyelitis: a 26-year mayo clinic experience. Clinical Infectious Diseases: an Official Publication of the Infectious Diseases Society of America, ciag048. Advance Online Publication. 2026; doi: 10.1093/cid/ciag048. PMC13341251 41637568

[22] Li YD, Wong CB, Tsai TT, et al. Appropriate duration of post-surgical intravenous antibiotic therapy for pyogenic spondylodiscitis. BMC Infect Dis. 2018;18(1):468. doi: 10.1186/s12879-018-3377-1. 30223785 PMC6142394

[23] Darouiche RO. Spinal epidural abscess. N Engl J Med. 2006;355(19):2012–2020. doi: 10.1056/NEJMra055111. 17093252

[24] Stratton A, Gustafson K, Thomas K, et al. Incidence and risk factors for failed medical management of spinal epidural abscess: a systematic review and meta-analysis. J Neurosurg Spine. 2017;26(1):81–89. doi: 10.3171/2016.6.SPINE151249. 27636865

[25] Kasliwal MK, Tan LA, Traynelis VC. Infection with spinal instrumentation: review of pathogenesis, diagnosis, prevention, and management. Surg Neurol Int. 2013;4(Suppl 5):S392–S403. doi: 10.4103/2152-7806.120783. 24340238 PMC3841941

[26] Palmowski Y, Bürger J, Kienzle A, et al. Antibiotic treatment of postoperative spinal implant infections. J Spine Surg. 2020;6(4):785–792. doi: 10.21037/jss-20-456. 33447684 PMC7797805

[27] Cho OH, Bae IG, Moon SM, et al. Therapeutic outcome of spinal implant infections caused by Staphylococcus aureus: a retrospective observational study. Medicine (Baltimore). 2018;97(40):e12629. doi: 10.1097/MD.0000000000012629. 30290637 PMC6200525

[28] Masters EA, Trombetta RP, de Mesy Bentley KL, et al. Evolving concepts in bone infection: redefining “biofilm”, “acute vs. chronic osteomyelitis”, “the immune proteome” and “local antibiotic therapy. Bone Res. 2019;7(1):20. doi: 10.1038/s41413-019-0061-z. 31646012 PMC6804538

[29] Kim D, Kim J, Kim T. Clinical characteristics of patients with pyogenic vertebral osteomyelitis and concurrent infections and their clinical outcomes. J Pers Med. 2022;12(4):541. doi: 10.3390/jpm12040541. 35455656 PMC9028400

[30] Li J, Zhao Y, Yang Y, et al. Development and validation of a nomogram for predicting mortality in patients with vertebral osteomyelitis. Infection. 2026; Advance online publication. doi: 10.1007/s15010-026-02741-x. 41758454

[31] Ziarko TP, Walter N, Schindler M, et al. Risk factors for the in-hospital mortality in pyogenic vertebral osteomyelitis: a cross-sectional study on 9753 patients. J Clin Med. 2023;12(14):4805. doi: 10.3390/jcm12144805. 37510920 PMC10381366

[32] Stærke NB, Nielsen RA, Kvist F, et al. Predictors of functional impairment after pyogenic vertebral osteomyelitis: a retrospective cohort study in the central Denmark region, 2017-2023. Int J Infect Dis. 2026;163:108191. doi: 10.1016/j.ijid.2025.108191. 41232751

[33] Balcescu C, Odeh K, Rosinski A, et al. High prevalence of multifocal spine infections involving the cervical and thoracic regions: a case for imaging the entire spine. Neurospine. 2019;16(4):756–763. doi: 10.14245/ns.1836296.148. 31284339 PMC6945002

[34] Gerstmeyer J, Gorbacheva A, Pierre C, et al. What is worse: a comparison of solitary versus multifocal pyogenic spondylodiscitis using a nationwide analysis of readmission rates and risk factors. J Clin Med. 2025;14(16):5784. doi: 10.3390/jcm14165784. 40869609 PMC12386682

[35] de Graeff JJ, Paulino Pereira NR, van Wulfften Palthe OD, et al. Prognostic factors for failure of antibiotic treatment in patients with osteomyelitis of the spine. Spine (Phila Pa 1976). 2017;42(17):1339–1346. doi: 10.1097/BRS.0000000000002084. 28134749

[36] Salvetti DJ, Tempel ZJ, Goldschmidt E, et al. Low preoperative serum prealbumin levels and the postoperative surgical site infection risk in elective spine surgery: a consecutive series. J Neurosurg Spine. 2018;29(5):549–552. doi: 10.3171/2018.3.SPINE171183. 30052149

[37] Gerstmeyer J, Pierre C, Schildhauer TA, et al. Malnutrition in spondylodiscitis: an overlooked risk factor. J Orthop Surg Res. 2025;20(1):17. doi: 10.1186/s13018-024-05431-2. 39773279 PMC11706169

[38] Salvetti DJ, Tempel ZJ, Gandhoke GS, et al. Preoperative prealbumin level as a risk factor for surgical site infection following elective spine surgery. Surg Neurol Int. 2015;6(Suppl 19):S500–S503. doi: 10.4103/2152-7806.166893. 26605112 PMC4617027

[39] Tempel Z, Grandhi R, Maserati M, et al. Prealbumin as a serum biomarker of impaired perioperative nutritional status and risk for surgical site infection after spine surgery. J Neurol Surg A Cent Eur Neurosurg. 2015;76(2):139–143. doi: 10.1055/s-0034-1394188. 25594818

[40] Kim J, Kang HS, Kim JW, et al. Treatment outcomes in patients with pyogenic vertebral osteomyelitis who have cirrhosis. Sci Rep. 2019;9(1):15223. doi: 10.1038/s41598-019-51758-w. 31645623 PMC6811580

[41] Akiyama T, Chikuda H, Yasunaga H, et al. Incidence and risk factors for mortality of vertebral osteomyelitis: a retrospective analysis using the Japanese diagnosis procedure combination database. BMJ Open. 2013;3(3):e002412. doi: 10.1136/bmjopen-2012-002412. PMC3612742 23533214

[42] Kernich N, Abi-Chokami A, Jung N, Interdisciplinary Studygroup of Spondylodiscitis – Cologne (IST-SPONDYL)., et al. Early and late mortality in vertebral osteomyelitis: who dies within the first year after diagnosis. Infection. 2025;53(5):2025–2035. doi: 10.1007/s15010-025-02541-9. 40343568 PMC12460364

[43] Schindler M, Walter N, Reinhard J, et al. Midterm survival and risk factor analysis in patients with pyogenic vertebral osteomyelitis: a retrospective study of 155 cases. Front Surg. 2024;11:1357318. doi: 10.3389/fsurg.2024.1357318. 38835852 PMC11148346

[44] Lee Y, Kim BJ, Kim SH, et al. Comparative analysis of spontaneous infectious spondylitis: pyogenic versus tuberculous. J Korean Neurosurg Soc. 2018;61(1):81–88. doi: 10.3340/jkns.2016.1212.005. 29354239 PMC5769839

[45] Lim JS, Kim TH. Recurrence rates and its associated factors after early spinal instrumentation for pyogenic spondylodiscitis: a nationwide cohort study of 2148 patients. J Clin Med. 2022;11(12):3356. doi: 10.3390/jcm11123356. 35743427 PMC9225581

[46] Kim J, Ryu H, Kim SW, et al. Prediction of recurrence in pyogenic vertebral osteomyelitis by artificial neural network using time-series data of C-reactive protein: a retrospective cohort study of 704 patients. Spine (Phila Pa 1976). 2021;46(18):1207–1217. doi: 10.1097/BRS.0000000000003985. 34435983

[47] Liu Y, Zheng Y, Ding S. Development and validation of a prognostic nomogram model for severe osteomyelitis patients. Sci Rep. 2025;15(1):318. doi: 10.1038/s41598-024-83418-z. 39747915 PMC11695742

[48] Zadran S, Pedersen PH, Eiskjær S. Vertebral osteomyelitis: a mortality analysis comparing surgical and conservative management. Global Spine J. 2020;10(4):456–463. doi: 10.1177/2192568219862213. 32435567 PMC7222680

